# Xanthine Oxidase Does Not Contribute to Apoptosis after Brain Hypoxia-Ischemia in Immature Rabbits

**DOI:** 10.1155/2013/253093

**Published:** 2013-08-01

**Authors:** Anthony Moretti, Alma Ramirez, Richard Mink

**Affiliations:** ^1^Division of Pediatric Critical Care Medicine, Department of Pediatrics, Harbor-UCLA Medical Center, Torrance, CA 90502, USA; ^2^Division of Pediatric Critical Care Medicine, Department of Pediatrics, Los Angeles BioMedical Research Institute, Torrance, CA 90502, USA; ^3^Division of Pediatric Critical Care Medicine, Department of Pediatrics, Harbor-UCLA Medical Center and Los Angeles BioMedical Research Institute, David Geffen School of Medicine at UCLA, Torrance, CA 90502, USA

## Abstract

*Background*. The mechanisms involving the initiation of apoptosis after brain hypoxia-ischemia through caspase activation are not fully defined. Oxygen free radicals may be an important mediator of caspase initiation with reactive oxygen species generated by xanthine oxidase (XO) being one potential source. The purpose of this study was to examine the role of XO in apoptosis after global cerebral injury. *Methods*. Immature rabbits were subjected to 8 minutes hypoxia and 8 minutes ischemia and then 4 hours of reperfusion. In one group (*n* = 5), the XO substrate xanthine was infused to generate more oxygen free radicals to promote apoptosis while in another (*n* = 5), the XO inhibitor allopurinol was given to reduce apoptosis by preventing free radical production (*n* = 5). Control animals (*n* = 4) received the vehicles. Caspase 3, 8, and 9 enzyme activities were measured in the cerebral cortex, hippocampus, cerebellum, thalamus, and caudate. *Results*. Administration of xanthine increased (*P* < 0.05) caspase 3 activity but only in the hippocampus, and pretreatment with allopurinol did not reduce it. No differences (*P* > 0.05) were found in any other region nor were there any changes in caspases 8 or 9 activities. *Conclusion*. We conclude that XO is not a major factor in inducing apoptosis after hypoxic-ischemic brain injury.

## 1. Introduction

The reperfusion of previously ischemic tissue is believed to contribute to injury through the generation of free radical species [1, 2]. During hypoxia, ATP is depleted and metabolized through various intermediates to hypoxanthine. When perfusion is reestablished, newly provided oxygen allows for the conversion of hypoxanthine to xanthine, and ultimately to uric acid, by the enzyme xanthine oxidase (XO) [1]. This metabolism of hypoxanthine and xanthine to uric acid by XO has been argued to be a source of oxygen free radicals causing cerebral injury [2–5]. However, in various animal models, inhibition of XO with allopurinol before ischemia has yielded inconclusive results [6–11].

Hypoxia-ischemia leads to not only necrosis but also apoptosis. Apoptosis is a highly ordered process of programmed cell death that can occur in both normal physiologic remodeling and pathologic processes [12]. This type of cell death can be recognized by several biochemical and morphological markers, such as chromatin condensation, DNA fragmentation, caspase activation, and mitochondrial alterations. Apoptosis may be the primary factor in the prolonged progression of neurodegeneration and cerebral dysfunction hours to days after injury [12, 13]. Evidence suggests that apoptosis after hypoxia-ischemia is triggered through the generation of free radicals [14]. These reactive oxygen species may be produced during reperfusion, at least in part, by XO [15]. 

Caspases play an important role in the initiation and execution of apoptosis [16]. These are a family of cysteine proteases that reside in the cytosol as inactive zymogens and become activated upon stimulation by proteolytic cleavage at internal aspartate residues. Two different classes of caspase enzymes are involved in the activation of the apoptotic pathway, one group in the early (initiator) phase (8, 9, and 10) and the other in the late (effector) phase (3, 6, and 7). Apoptosis-mediated cell death triggers initiator caspases that in turn activate effector caspases in a self-amplifying cascade.

No previous study has investigated the contribution of XO in apoptosis-mediated cell death in hypoxic-ischemic brain injury. Further understanding of the mechanism of this injury could provide valuable therapeutic targets to limit ongoing brain damage. Our hypothesis was that XO contributes to apoptosis-mediated cell death after hypoxia-ischemia brain injury. We attempted to promote free radical generation and thereby apoptosis-mediated cell death by infusing xanthine, an XO substrate. In another approach, we reduced XO free radical generation by administering allopurinol, a specific inhibitor of XO, to decrease apoptosis. We compared caspase 3, 8, and 9 activities four hours after hypoxia-ischemia injury and reperfusion in the brains of immature rabbits pretreated with allopurinol, with rabbits infused with xanthine, and with those infused with the vehicle. We also measured caspase 3, 8, and 9 activities among various brain regions in rabbits subjected to hypoxic-ischemic brain injury to determine regional variations in enzyme activities.

## 2. Materials and Methods

### 2.1. Animal Preparation

The study utilized a rabbit model of global cerebral hypoxia-ischemia previously reported by Mink and Johnston [8] and was approved by the Institutional Animal Use and Care Review Committee. Immature New Zealand white rabbits weighing between 1.0 and 1.4 kilograms were initially anesthetized with 7% sevoflurane or 5% isoflurane and then maintained on 2% sevoflurane or 1% isoflurane, respectively. A tracheostomy was performed, and the rabbits were intubated and mechanically ventilated. Catheters were placed in both femoral arteries and veins by cutdown for continuous monitoring of blood pressure, to obtain blood samples and to administer medications. The scalp was exposed, and a Camino fiberoptic catheter was placed to monitor intracranial pressure (ICP). An epidural needle was inserted into the subarachnoid space for infusion of artificial cerebrospinal fluid (CSF). Intraparenchymal brain temperature was monitored by a thermocouple temperature sensor inserted into the frontal lobe, and an esophageal temperature probe was placed to record body temperature. 

### 2.2. Inducing Brain Injury

Eight minutes of cerebral hypoxia was induced by ventilating the rabbit with a gas mixture of 4% oxygen, 4% carbon dioxide, and 92% nitrogen. Immediately following 8 minutes of hypoxia, cerebral ischemia was achieved by infusing warmed, artificial CSF solution [17], such that mean ICP exceeded mean arterial pressure (MAP). Brain and esophageal temperatures were maintained at the normal rabbit values of 38.5 to 39.5°C during the experiment with a heating blanket and an infrared heating lamp and by surrounding the head with warmed, humidified air. After 8 minutes of ischemia, reperfusion was initiated by allowing the CSF to drain until ICP was <20 mmHg. Blood pressure was supported with fluid boluses of normal saline or an epinephrine infusion, as needed, to maintain a cerebral perfusion pressure (CPP) of at least 50 mmHg. After verifying adequate anesthesia, the animals were killed after 4 hours of reperfusion with an infusion of saturated potassium chloride. The calvarium was resected, and the brain was immediately removed and chilled in liquid nitrogen. Sections of the cerebral cortex, hippocampus, thalamus, caudate, and cerebellum were frozen at −80°C for later assay of caspase activity and for measurement of the amount of xanthine and allopurinol in the cerebral cortex. Arterial blood gases and hematocrit were measured using the iSTAT portable clinical analyzer (Abbott Point of Care, Princeton, NJ).

### 2.3. Experimental Groups

Rabbits were randomly assigned to either control (CTL), allopurinol (ALLO), or xanthine group (XAN). All animals were exposed to hypoxic-ischemic brain injury. In the XAN group, rabbits received an xanthine infusion of 600 *μ*mole per kilogram per hour mixed in 1/4 normal saline at pH 8.7 starting 30 minutes before injury and continuing through the first 30 minutes of reperfusion. This dose was used in a previous experiment and was shown to increase brain xanthine levels [8]. In the ALLO group, rabbits received allopurinol 100 mg/kg (mixed in 1/4 normal saline, pH 11.3) i.p. daily for 2 days prior to the experiment. On the day of the experiment, rabbits in the ALLO group received an additional 100 mg/kg dose i.v. Animals that did not receive allopurinol or xanthine received an equivalent volume of the appropriate vehicle. 

### 2.4. Caspase Activity Assays

Caspase activities were determined using a modification of commercially available kits (BioVision Research Products, Mountain View, CA). Brain tissue was homogenized in Cell Lysis Buffer (BioVision Research Products, Mountain View, CA) using a Tissue Tearor (Bio Spec Products, Racine, WI) at a speed of 3 for 35 seconds. Samples were centrifuged at 25,000 g × 60 min at 4°C, and the supernatant was removed. 50 *μ*L of supernatant was mixed with 50 *μ*L 2X reaction buffer/DTT and 5 mL substrate (BioVision Research Products, Mountain View, CA), and the change in absorbance was measured at 405 nm at 37°C in a microplate reader (BioTek, Winooski, VT) for two hours. Data from 60 to 120 minutes were used in the analysis. Assays were performed in triplicate, the samples blank corrected, and the results averaged. A regression equation was created in which activity was represented by the slope of the equation. 

To generate a standard enzyme curve, various dilutions of known caspase enzyme (caspase 3: product number 1083-100; caspase 8: 1088-100; caspase 9: 1089-100; all supplied by BioVision Research Products, Mountain View, CA) were assayed as described earlier. For each dilution, a regression equation was created. Then, the slopes of the known enzyme activities were used to develop a standard curve. The caspase activity in the brain tissue was calculated using the standard enzyme curve. Protein concentration of supernatant was determined using the Pierce BCA Protein Assay (Thermo Fisher Scientific Inc., Rockford, IL). Activity is expressed as units per milligram of protein where a unit is defined as the amount converted in nanomoles per hour. 

To confirm specificity of the assay, supernatants were incubated with specific caspase inhibitors and the percent reduction of activity determined. Incubation with the specific caspase 8 inhibitor ZIETD-FMK (BioVision Research Products, Mountain View, CA) reduced activity by 97% while incubation with Z-DEVD-FMK (BioVision Research Products, Mountain View, CA) for caspase 3 and Z-LEHD-FMK (BioVision Research Products, Mountain View, CA) for caspase 9 resulted in 100% and 95% reductions in activity, respectively. The coefficient of variation for caspase 8 assay was 13.8% while that for caspase 9 was 7.7% and for caspase 3 was 21.9%. 

### 2.5. Measurement of Brain Xanthine and Allopurinol

Cerebral cortical tissue was homogenized in ice-cold 1.3 M perchloric acid and after a 30-minute period to allow for extraction, centrifuged at 15,000 g × 15 minutes at 4°C. The supernatant was removed, and the pH was adjusted to 7.3–7.5 with 6 M potassium hydroxide/1 M potassium carbonate. The sample was then centrifuged at 14,900 g × 10 minutes at 4°C, and the supernatant was removed. Subsequently, the amount of xanthine and allopurinol was determined by HPLC with detection at 254 nm. For xanthine, a 50 *μ*L aliquot was separated on a 3.9 mm ID × 300 mm Bondapak C18 125 Å 10 *μ*m column (Waters, Milford, MA) with a mobile phase of 10 mM ammonium phosphate in acetonitrile (10 : 1, v/v), pH 5.5, at 1 mL/minute. Measurement of allopurinol utilized a 75 *μ*L injection with a 4.6 mm ID × 100 mm HyperClone 3 *μ*m ODS C18 120 Å column (Phenomenex, Torrance, CA) and a mobile phase of 66 mM disodium phosphate/66 mM sodium biphosphate, pH 5.5 at 0.8 mL/minute. Assays were performed in duplicate, and the results were averaged. Values for the sample were calculated from peak areas using known standards.

### 2.6. Statistical Analysis

Caspase 3, 8, and 9 activities were compared among the groups and in each brain region with ANOVA. This was also used to analyze brain xanthine levels. Post hoc testing utilized Fisher's LSD. Physiologic and blood gas variables were analyzed using repeated-measures ANOVA. The data are expressed as mean ± SD. *P* < 0.05 was considered statistically significant.

## 3. Results

A total of 14 rabbits were evaluated in the study, 4 in the CTL group, 5 in ALLO, and 5 in XAN. The animal weights did not differ (*P* > 0.05) between the 3 groups (CTL: 1.37 kg ±0.01, XAN: 1.34 ± 0.09, ALLO: 1.37 ± 0.15). The time required for removal of the brain to placement in liquid nitrogen also did not differ (*P* > 0.05; CTL 145 s ± 37, XAN 124 ± 21, ALLO 131 ± 12).

### 3.1. Physiological Data

Physiological data are displayed in Table 1 and arterial blood gas data in Table 2. There were no significant differences (*P* > 0.05) in baseline physiologic data or arterial blood gas values. Mean CPP during hypoxia was >50 mmHg in all animals and greater than 60 mmHg during reperfusion. As expected, mean ICP exceeded MAP during ischemia resulting in a CPP of 0. Brain temperature and esophageal temperatures were maintained between 38.5°C and 39.5°C throughout the experiment. No significant acidemia (pH < 7.15) was noted in any rabbit. Mean pO_2_ during hypoxia was <25 mmHg but by 30 minutes of reperfusion, oxygen levels had returned to baseline and remained so throughout the 240 minutes of reperfusion.

### 3.2. Brain Xanthine and Allopurinol Levels

**Figure 1:**
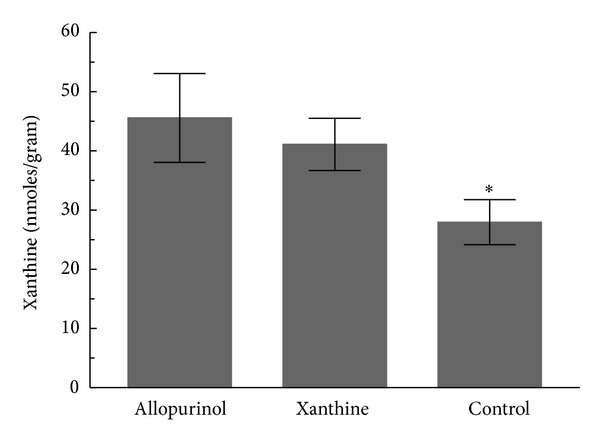
The amount of xanthine (nmoles/gram) in the cerebral cortex after hypoxia-ischemia and 4 hours of reperfusion (mean ± SD). *P < 0.05 versus other groups.

The amount of xanthine in the cerebral cortex was higher in rabbits that received the xanthine infusion or allopurinol compared with those that were administered the vehicle (Figure 1, *P* < 0.05). In the ALLO animals, the amount of the allopurinol was 4.9 ± 2.0 *μ*M with the lowest value in any rabbit 2.9 *μ*M.

### 3.3. Caspase 3 Activity

**Figure 2:**
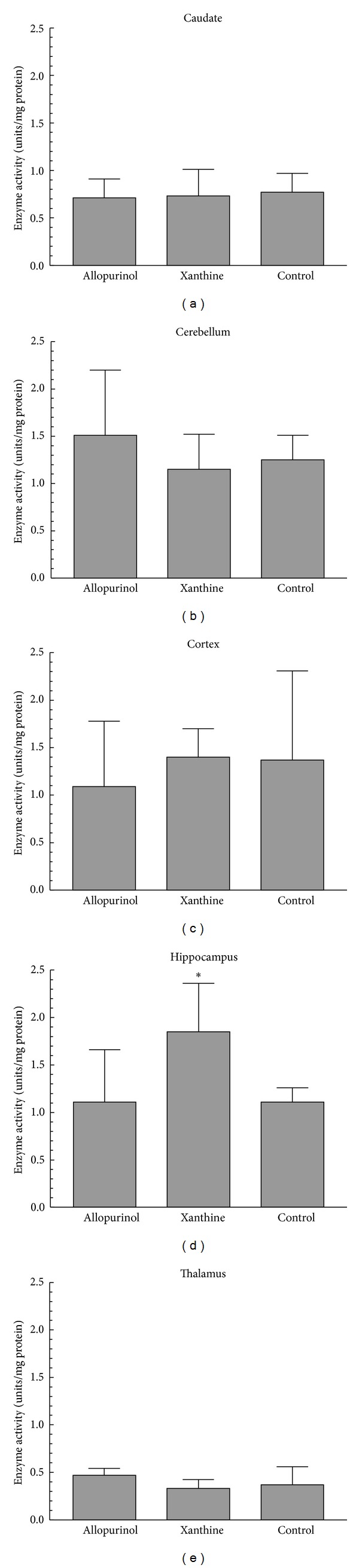
Caspase 3 enzyme activity in the different brain regions after hypoxia-ischemia and 4 hours of reperfusion (mean ± SD). *P < 0.05 versus other groups.

Compared with the other groups, caspase 3 activity in the XAN animals was higher in the hippocampus (Figure 2, *P* < 0.05). There were no differences (*P* > 0.05) in any of the other regions. However, there were regional differences in caspase 3 activity. Activity was the greatest (*P* < 0.05) in the hippocampus (1.27 ± 0.04 units/milligram protein), cortex (1.31 ± 0.32), and cerebellum (1.31 ± 0.25) when compared with the caudate (0.74 ± 0.08) and thalamus (0.39 ± 0.07). 

### 3.4. Caspase 8 Activity

**Figure 3:**
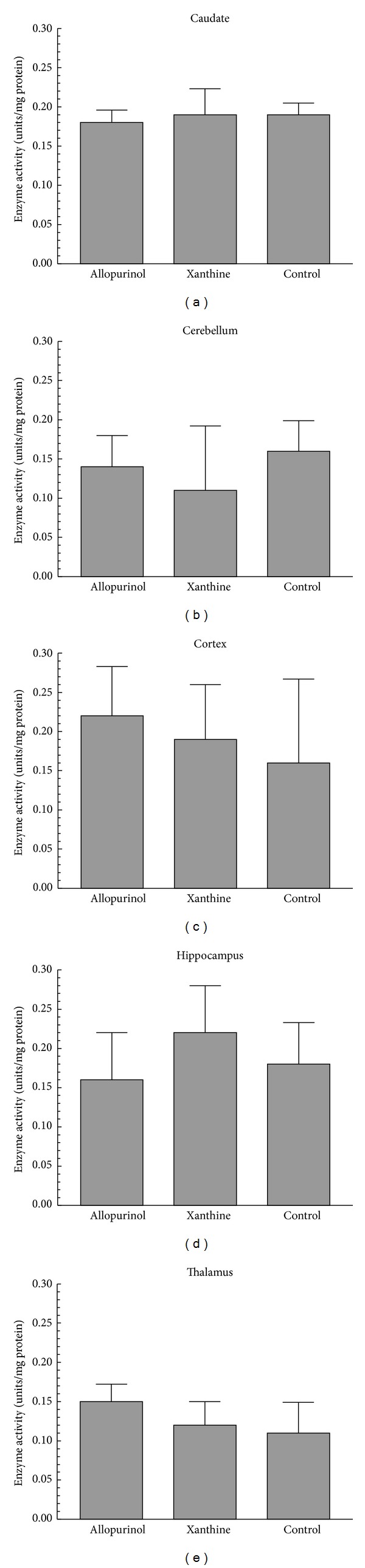
Caspase 8 enzyme activity in the various brain regions after hypoxia-ischemia and reperfusion (mean ± SD). There was no difference (*P* > 0.05) among the groups.

For caspase 8, no differences (*P* > 0.05) were found among the groups (Figure 3). When compared with caspases 3 and 9, overall caspase 8 activity was low, ranging from 0.14 to 0.22 units per milligram protein. The greatest amount (*P* < 0.05) of caspase 8 activity was observed in the hippocampus (0.32 units/milligram protein ± 0.32) compared with the other regions (cortex: 0.21 ± 0.05, cerebellum: 0.14 ± 0.04, caudate: 0.19 ± 0.02 and thalamus: 0.13 ± 0.03).

### 3.5. Caspase 9 Activity

**Figure 4:**
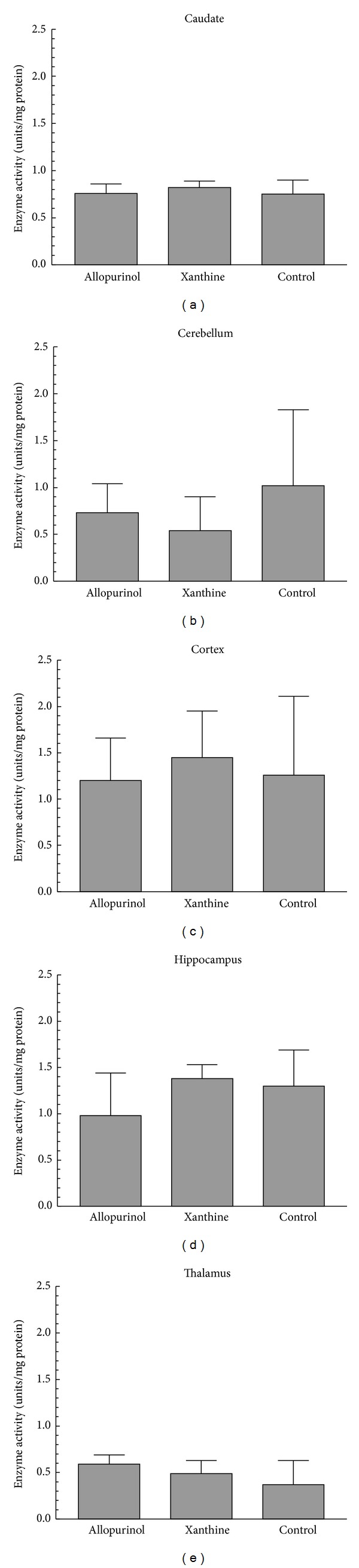
Caspase 9 enzyme activity in the brain regions (mean ± SD). There was no difference (*P* > 0.05) among the groups.

Similar to caspase 8, no group differences (*P* > 0.05) were found for caspase 9 (Figure 4). However, as opposed to that of the other caspases, the highest amount (*P* > 0.05) of caspase 9 activity was observed in the cortex (1.45 units/milligram protein ± 0.39) followed by that in the hippocampus (1.15 ± 0.39), activity of which was also greater (*P* < 0.05) than that in the other regions (cerebellum: 0.74 ± 0.50; caudate: 0.78 ± 0.10; thalamus 0.54 ± 0.11).

## 4. Discussion

Free radicals formed after hypoxic-ischemic injury are thought to induce apoptosis. In one proposal, purines metabolized by XO during hypoxia-ischemia lead to the conversion of hypoxanthine to xanthine and uric acid producing free radicals [14]. By inhibiting XO activity with allopurinol, free radical generation should be reduced. However, inhibition of XO also leads to enhanced purine recovery and increased restoration of high-energy phosphates [18]. Consequently, the effects of XO inhibition cannot be solely attributed to a reduction in free radical generation. To better elucidate the role of XO in apoptosis, we used two approaches, an xanthine infusion to increase apoptosis and an XO inhibitor to reduce it. By providing a substrate or an inhibitor of XO, we expected to see an increase in caspase enzyme activation in rabbits infused with xanthine and a decrease in those administered allopurinol. We did detect an increase in caspase 3 activity in hippocampus in the xanthine infused animals, but no change with administration of allopurinol. We did not find differences in any other brain region. We believe that these data do not support a role for XO generated free radicals in contributing to apoptosis after brain hypoxia-ischemic injury.

Two other investigators have evaluated the effect of XO inhibition on apoptosis. Peeters-Scholte et al. [19] subjected piglets to 1 hour of cerebral hypoxia-ischemia and then administered allopurinol immediately upon reperfusion. At 24 hours postinjury, there were no differences in caspase 3 activity, histopathology or TUNEL-labeling in the cortex, hippocampus or striatum between the allopurinol and control groups. However, these investigators infused the allopurinol after injury, and it is unclear that XO was inhibited in the early phase of reperfusion when free radical generation is maximal [20, 21]. In an *in vitro* investigation utilizing rat hepatocytes, Herrera et al. [22] examined the role of several catalytic enzymes that could generate reactive oxygen species and promote apoptosis. These included NAPDH oxidase, NADH dehydrogenase, cytochrome P450, cyclooxygenase, and XO. Only inhibition of NADPH oxidase produced a reduction of caspase 3 activation. Inhibition of XO with allopurinol had no effect. These findings are in agreement with our data that support a minimal role of XO in contributing to apoptosis after cerebral hypoxia-ischemia.

We measured caspase activities only 4 hours after injury, consistent with a time in which others have reported apoptotic changes. In a study by Khurana et al. [23], newborn piglets were subjected to hypoxic conditions for 1 hour, and caspase protein and activity were measured in the cerebral cortex. After only one hour following the injury, caspases 3, 8, and 9 enzyme proteins and activities were significantly elevated in the hypoxic group compared with nonhypoxic controls. In another piglet model of cerebral ischemia, Pirzadeh et al. [24] reported an increase in Bax, a neuroregulatory protein that promotes apoptosis, as early as 2 hours after injury in the cortex, hippocampus, and striatum. 

We confirmed that infusion of xanthine increased the amount of xanthine in the brain. High levels of allopurinol were also detected. The lowest concentration of allopurinol was 2.9 *μ*M, much greater than the reported IC_50_ of 0.8 *μ*M for inhibition of XO [25]. In addition, we noted that the amount of xanthine in the brains of rabbits who received allopurinol was higher than that in controls. Xanthine is generated from two sources, conversion from hypoxanthine by XO and deamination of guanine by guanase. It is subsequently metabolized to uric acid by XO. Since allopurinol inhibits XO but would not alter guanase activity, the accumulation of xanthine in the ALLO animals provides evidence that XO was inhibited, since xanthine formed from guanine would not be able to be converted to uric acid. Elevation of brain guanine has been reported after cerebral ischemia and reperfusion [26].

Infusion of xanthine increased caspase 3 activity in the hippocampus but pretreatment with allopurinol did not reduce activity of this enzyme. In addition, the effect with xanthine administration was not seen with either of the initiator caspases, 8 or 9. If XO does not contribute to apoptosis, why did the xanthine infusion increase caspase 3 activity in the hippocampus? It is possible that the xanthine served as substrate for another enzyme that produced free radicals that would not be affected by allopurinol. Although it is not a preferred substrate, aldehyde oxidase will convert xanthine to uric acid and generate hydrogen peroxide in the process [27]. Since the hippocampus is thought to be a particularly vulnerable area, this effect might not have been observed in other regions [28]. 

There were regional differences in the amount of the caspase enzymes. For the initiator and effector caspases, activity was high in the hippocampus and cerebral cortex. This is consistent with the vulnerability of these regions to hypoxic-ischemic injury. However, based on the ratio of Bcl-2/Bax and compared with the cortex and hippocampus, Pirzadeh and colleagues suggested that the striatum was the most susceptible area [24].

We used a novel kinetic assay to measure caspase enzyme activity. Rather than to measure activity relative to controls, we used caspases of known activities to generate a standard curve and utilized this to determine absolute enzyme activity in the samples. To our knowledge, this is the first kinetic caspase assay to provide this measure of activity. Using specific caspase inhibitors, we showed that the assays were specific and confirmed that the coefficient of variation for each of the caspase assays was excellent. 

The sample size in our investigation was small but we believe that the study was adequately powered. In a study of similar design to this one, Khurana et al. [23] reported a 60% increase in caspase 3 activity, a 57% elevation in caspase 8, and a 31% increase in caspase 9 when compared with noninjured animals. A posthoc power analysis of the data in our investigation revealed that the power was 80% (*α* = 0.05) to detect a 67% difference in caspase 3 activity, a 40% difference in caspase 8, and a 55% change in caspase 9. Nonetheless, there are other factors that must be considered. Apoptosis is a highly complex physiological process involving several converging pathways. We evaluated only 3 enzymes involved in the apoptotic cascade. We also did not examine other markers of apoptosis and cell death such as BCL or TUNNEL. In addition, we measured caspase activity after 4 hours of reperfusion.

We found that xanthine infusion increased caspase 3 activity but only in the hippocampus, and pretreatment with allopurinol did not reduce it. No differences were found in any other regions or for caspases 8 or 9. These data do not support a role for XO in contributing to apoptosis after brain hypoxic-ischemic injury.

## Figures and Tables

**Table 1:** Physiologic parameters during cerebral hypoxia-ischemia and reperfusion (mean ± SD).

	Baseline	Hypoxia	Ischemia	Reperfusion	Reperfusion	Reperfusion
		7 minutes	7 minutes	30 minutes	60 minutes	240 minutes
Mean arterial pressure (mmHg)						
Control	91 ± 9	106 ± 20	88 ± 15	87 ± 4	85 ± 5	77 ± 33
Xanthine	95 ± 5	102 ± 11	94 ± 10	95 ± 6	90 ± 3	97 ± 12
Allopurinol	100 ± 7	94 ± 17	96 ± 4	95 ± 19	86 ± 9	85 ± 4
Intracranial pressure (mmHg)						
Control	5 ± 3	13 ± 8	100 ± 17	5 ± 4	6 ± 5	1 ± 2
Xanthine	1 ± 1	10 ± 6	109 ± 8	3 ± 2	5 ± 3	2 ± 3
Allopurinol	2 ± 2	9 ± 2	107 ± 4	3 ± 2	4 ± 4	2 ± 2
Cerebral perfusion pressure (mmHg)						
Control	87 ± 7	93 ± 14	0 ± 0	82 ± 7	79 ± 6	76 ± 32
Xanthine	94 ± 5	92 ± 10	0 ± 0	93 ± 4	86 ± 2	95 ± 10
Allopurinol	98 ± 8	84 ± 16	0 ± 0	93 ± 18	82 ± 9	83 ± 3
Esophageal temperature (°C)						
Control	39.8 ± 0.7	39.7 ± 0.2	40.0 ± 0.2	39.8 ± 0.7	39.9 ± 0.4	39.5 ± 0.2
Xanthine	39.9 ± 0.1	39.4 ± 0.2	39.8 ± 0.1	39.7 ± 0.3	39.9 ± 0.4	39.3 ± 0.1
Allopurinol	39.8 ± 0.6	39.4 ± 0.1	39.9 ± 0.3	39.5 ± 0.5	39.9 ± 0.7	39.5 ± 0.2
Brain temperature (°C)						
Control	39.3 ± 0.3	39.3 ± 0.2	39.0 ± 0.2	38.9 ± 0.5	39.2 ± 0.3	38.4 ± 0.6
Xanthine	39.1 ± 0.3	38.9 ± 0.1	38.9 ± 0.3	38.8 ± 0.4	39.3 ± 0.4	38.6 ± 0.1
Allopurinol	39.1 ± 0.3	38.9 ± 0.1	39.1 ± 0.3	38.6 ± 0.4	39.3 ± 0.5	38.7 ± 0.0

**Table 2:** Blood gas values and hematocrit during cerebral hypoxia-ischemia and reperfusion (mean ± SD).

	Baseline	Hypoxia	Reperfusion	Reperfusion	Reperfusion
		7 minutes	30 minutes	60 minutes	240 minutes
pH					
Allopurinol	7.46 ± 0.04	7.24 ± 0.07	7.28 + 0.06	7.36 ± 0.11	7.42 ± 0.06
Xanthine	7.51 ± 0.08	7.33 ± 0.06	7.30 ± 0.06	7.40 ± 0.04	7.29 ± 0.07
Control	7.48 ± 0.06	7.30 ± 0.06	7.30 ± 0.06	7.36 ± 0.07	7.41 ± 0.07
pCO_2_ (mmHg)					
Allopurinol	22 ± 2	34 ± 2	25 ± 3	26 ± 3	23 ± 2
Xanthine	24 ± 4	33 ± 3	27 ± 3	25 ± 3	26 ± 3
Control	24 ± 5	33 ± 3	27 ± 1	28 ± 3	24 ± 3
pO_2_ (mmHg)					
Allopurinol	101 ± 32	19 ± 1	119 ± 47	97 ± 15	92 ± 12
Xanthine	107 ± 22	19 ± 2	89 ± 8	91 ± 12	72 ± 16
Control	90 ± 17	20 ± 2	76 ± 19	76 ± 25	90 ± 10
Hematocrit (%)					
Allopurinol	27 ± 5	28 ± 3	23 ± 6	27 ± 4	26 ± 3
Xanthine	28 ± 2	27 ± 4	27 ± 3	28 ± 1	28 ± 8
Control	25 ± 3	26 ± 2	25 ± 5	26 ± 3	20 ± 8
